# Exploring the acceptance of mozzarella cheese in school lunches among school-aged children: a pilot study

**DOI:** 10.3389/fnut.2025.1495180

**Published:** 2025-01-24

**Authors:** Xiaofang Lin, Qin Zhang, Qiong Wu, Wenxuan Huo, Xiaoying Zhang, Shanshan Geng, Yizhou Lv, Yuanyuan Li, Yilun Wang, Linjing Dong, Xue Leng, Zhenghao Lin, Ignatius Man-Yau Szeto, Zhixu Wang

**Affiliations:** ^1^Department of Maternal, Child and Adolescent Health, School of Public Health, Nanjing Medical University, Nanjing, China; ^2^Nanjing Municipal Healthcare Center for Primary and Secondary Schools, Nanjing, China; ^3^Experimental Primary School, Nanjing Agricultural University, Nanjing, China; ^4^Innovation Centre, Inner Mongolia Yili Industrial Group Co., Ltd., Hohhot, China; ^5^National Center of Technology Innovation for Dairy, Hohhot, China

**Keywords:** cheese, school lunch, acceptability, familiarity, food pairing, traditional Chinese cooking methods

## Abstract

**Introduction:**

Dairy products are an important source of high-quality protein, calcium, vitamin A, and other nutrients, and they are a crucial part of a balanced diet for children. However, the daily intake of dairy products among school-age children in China is significantly inadequate. Considering school lunches as a vital pathway for children to obtain nutrients, this study aims to develop school lunch dishes incorporating mozzarella cheese and to assess the acceptance of these dishes among school-age children.

**Methods:**

This study was carried out in a primary school which has a self-run canteen in Nanjing. We innovatively integrated cheese with traditional Chinese food, and conducted a 3-month pilot study to develop dishes that meet the nutritional needs and sensory experience of students. 121 students with an average age of 9.8 years were invited to assess each dishes’ appearance, aroma, taste and overall liking, by using a 5-point Likert Scale. Focus group discussions were conducted after the project to further discover students’ attitude toward cheese dishes and canteen cooks’ experience in improving cheese dishes.

**Results:**

During the program, 16 cheese dishes were made, including 3 steamed dishes, 2 ready-to-eat foods (only heating required), and 11 stir-fried dishes. The overall liking’ results showed that ready-to-eat foods were the most popular among students, and steamed dishes ranked higher than stir-fried dishes (*P* < 0.001). Among the stir-fried dishes, students’ liking scores differed for cheese dishes made from different raw materials, pure meat food was more popular than vegetable food (*P* = 0.003), meat and vegetable food (*P* = 0.012). Additionally, focus group discussions found that students gave more positive ratings to and ready-to-eat foods and steamed dishes.

**Conclusion:**

Cheese can be well combined with traditional Chinese ingredients and be accepted, especially steamed or combined with meat. Introducing cheese in school lunches not only helps cultivate a habit of consuming cheese among children and adolescents from a young age, but also aids in closing the gap between their dairy consumption and dietary guidelines.

## 1 Introduction

School lunch has a vital role in the lives of school students. It not only fulfills their nutritional requirements to promote growth and development but also ensures optimal learning efficiency (1). However, the results of school lunch surveys in several cities in China reveal a common issue: inadequate provision of dairy products in school lunch has led to insufficient calcium intake (2–4). Data from the 2016–2017 China Child and Lactating Mother Nutrition and Health Surveillance dietary survey showed that children and adolescents aged 6–17 years consumed an average of 62.62 g of milk daily. This is significantly lower than the recommended amount which was 300 g of milk in the Chinese Dietary Guidelines for school-age children. Only 2.86% of children and adolescents have met the recommended intake (5). Therefore, it is imperative to promptly address the insufficient availability of dairy products in school lunches.

Cheese is a dairy product made by fermenting concentrated milk and removing whey. Cheese is a small-sized and highly nutritious food, rich in proteins and calcium. It boasts a higher nutrient density of these key nutrients compared to other dairy products. Moreover, the protein in cheese is primarily in the form of casein, which breaks down into smaller molecules such as amino acids and peptides during the fermentation process of cheese production, thus enhances the absorption rate in the body (6). Additionally, the presence of a diverse microbiota in mature cheese can alter the overall composition of oral flora and help to prevent tooth decay and cavities (7). Some studies suggest that the moderate cheese intake can reduce the risk of overweight or obesity, hypertension, and type 2 diabetes mellitus, with fresh cheese showing particularly significant effects (8–10). In China, the incidence of lactase deficiency was 87.6% in children aged 7–8 years and 87.8% in children aged 11–13 years. The incidence of lactose intolerance among school-age children in China is higher than 30% (11). However, during the cheese production process, a significant amount of lactose is eliminated along with the whey, whereas the remaining portion undergoes fermentation to convert into lactic acid, making cheese a nutritious food choice for individuals who are lactose intolerant (12). Therefore, incorporating cheese into the Chinese diet along with other foods can enhance the nutritional completeness and bolster the sustainability of the school meal system.

Currently, the predominant consumption of dairy products in China is in the form of liquid milk, with cheese accounting for less than 1% of overall consumption (13). At the same time, owing to food globalization and economic development, Chinese consumers have been steadily increasing their cheese consumption in recent years by eating in Western fast-food chains, such as Kentucky Fried Chicken. This indicates the market for cheese in China is highly promising with significant growth potential. However, a singular form of consumption is not enough to meet the diverse demands of the masses. In order to further promote cheese consumption, it is crucial to create more diversified consumption scenarios, such as incorporating cheese into family dinner tables. Research conducted by Rong Yi et al. on 1,260 young consumers nationwide revealed that nearly 50% of the participants expressed a desire for cheese that aligns with Chinese tastes and preferences, and approximately 70% of the surveyed consumers demonstrated interest and willingness to purchase cheese that can be paired with traditional Chinese foods, including noodles, steamed buns, rice, and various Chinese dishes (14).

For most Chinese people, cheese is still regarded as an exotic delicacy that poses challenges when incorporated into recipes. However, Japan and the Republic of Korea, both of which do not have a traditional culinary culture centered around cheese, have successfully incorporated cheese into their local cuisines. In Japan, cheese is commonly used in sushi and seafood dishes, and the Republic of Korea incorporates cheese into various dishes such as cheese ribs, cheese hot pot, and cheese rice cakes. This localization has led to a significant increase in cheese consumption in both countries, making them the highest consumers of cheese in Asia (15, 16). Utilizing the successful localization strategies employed by Japan and the Republic of Korea could provide an effective framework for China to promote cheese consumption within its own market.

In the initial phase of introducing cheese into school lunches in China, it is strategic to select types of cheese that are already familiar to the Chinese people. Mozzarella, representing fresh and curd-type cheeses, is the main type of cheese imported in China, accounting for over 50% of the market share (17). In addition, Mozzarella cheese is ideal for cooking due to its ability to shred and melt and its appealing stretch. Chinese consumers generally prefer soft and mild cheeses to more pungent varieties. Moreover, incorporating cheese into traditional Chinese cooking is still a novel food for Chinese children. Sensory characteristics play a significant role in children’s acceptance of new foods, and in the process of creating cheese-based Chinese meals, it is crucial to understand participants’ opinions on the appearance, aroma, and taste of these dishes (18).

The objectives of this study were twofold: (1) to incorporate mozzarella cheese into school lunch and to craft a diverse range of cheese-based meals; and (2) to evaluate the acceptability (appearance, aroma, taste and overall liking) of created school meals with cheese among school-age children, with the goal of introducing cheese into school lunches to improve the insufficient dairy intake of children in the further.

## 2 Materials and methods

### 2.1 Creation of the Chinese cheese dishes

To spread consumption throughout the school canteens and address the consumer barrier of unfamiliarity with how to prepare cheese, 16 initial recipes were provided by the elementary school chef in a school canteen. Subsequently, 2 professional nutritionists revised the ingredients and food pairing to form recommended weekly menus of school lunches. A total of 12 weekly menus were provided in the study, each including five lunch recipes, with three of those recipes containing cheese dishes. In this 12-week intervention, Chinese dishes with cheese were served to all children three times per week. Each student was offered 35 g of cheese per serving, as much as possible. The nutrient reference standards for the weekly menus of school lunches were based on the Nutrition Guidelines of School Meals (WS/T 554-2017) and Dietary Guidelines for Chinese Residents (2022). Supplementary Table 1 presents the lunch recipes for primary school students in the first week of the study.

The recipes emphasized the versatility of cheese, including applications such as:

• Combining cheese and meat, vegetable, or both.
• Processed using steaming or frying, which are the only 2 cooking methods in the general school canteens.
• Simple, quick recipes such as cheese rice cakes (ready-to-cook foods).

### 2.2 Cooking processes

**FIGURE 1:**
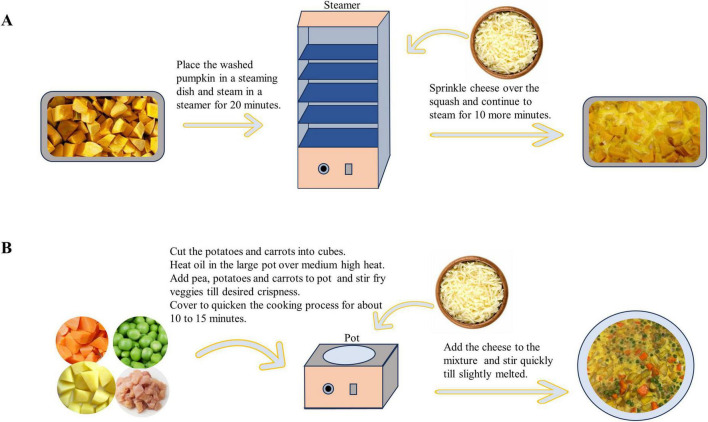
The cooking processes of two cheese dishes. **(A)** The cooking process of steamed pumpkin with cheese; **(B)** the cooking process of stir-fried vegetable cubes with cheese.

Mozzarella cheese, which is the only cheese product we use, with a protein content of 20.0 g/100 g and 630 mg/100 g of calcium, was obtained from Inner Mongolia Yili Industrial Group Co., Ltd., and vacuum-packed and stored at −20°C if not immediately utilized. Prior to use, samples were left in a refrigerator at 2–4°C for 24 h to thaw. The dishes were cooked according to the recipe requirements without cheese. Cheese was added to the mixtures a few minutes before the dishes were ready and cooked until the cheese was slightly melted and stirred quickly to prevent the cheese from sticking to the bottom of the pan. As an illustration, Figure 1 depicts the cooking processes of two cheese dishes called “Steamed Pumpkin with Cheese” and “Stir-Fried Vegetable Cubes with Cheese.”

### 2.3 Consumer panels

This pilot study, conducted between September 2022 and December 2022, utilized a cluster sampling method to select all students from three out of six classes in grade 4 at an elementary school in Nanjing. Nanjing is the capital of Jiangsu Province and is one of the more economically developed cities in China, with a total of 411 primary schools. The primary school involved in the study was randomly selected through a lottery system. Information on the enrolled children was obtained by sending online questionnaires to the students’ parents.

The sample size was determined using the following formula:$n=\frac{Z_{\propto }^{2}\,\left(1-P\right)}{\varepsilon^{2}\,P}$, where Z denotes the critical value for a 95% confidence level (Z_α_ = 1.96), ε represents the maximum relative error (10%), and *P* represents the prevalence of liking cheese dishes among the target population, which is estimated to be 80% based on a nationwide study in China (14). This calculation indicated a minimum requirement of 96 participants. Given a dropout rate of 20%, our study enrolled a total of 121 students [58 girls, 63 boys; average age 9.8 years, standard deviation (Std dev) 0.3 years], meeting the target sample size.

All children participated voluntarily with their parent’s or legal guardian’s informed written consent. The study was conducted in accordance with the Declaration of Helsinki, and approved by the Ethics Committee of Nanjing Medical University on 6 January 2022. The ethical approval project identification code is 2022-664.

### 2.4 Acceptance measurements

Initially, the teacher of the class informed students that their lunch for the day would include cheese dishes and encouraged them to eat as much as possible. Only one cheese dish was served during each lunch session. Second, children were given a paper score sheet to express their overall liking and liking for a series of sensory dimensions, including appearance, aroma, and taste using a 5-point hedonic scale from 1 (extremely dislike) to 5 (extremely like) (19). In addition, children were asked to answer the question “if they would recommend their parents to buy it or make it at home” (Yes/No). All necessary materials were set up in the classrooms where the children usually eat. Lunch was served on plates in the same quantities to each student. The children were allowed to sit in their regular seats without being separated. The tasting sessions were conducted between 11:30 AM and 12:30 PM at school, lasting 45–60 min. To minimize the influence of peers, a teacher and two researchers organized discipline in the class during the tasting process, ensuring that the children remained as quiet as possible and did not communicate with each other.

### 2.5 Focus group discussion

Two separate focus group discussions were conducted in combination with the hedonic product testing. A focus group discussion was conducted with a subgroup of children who participated in the hedonic test (*N* = 12), and another was held with the chef’s staff participants (*N* = 4). The meetings were conducted post-intervention, each lasting approximately one and a half hours, and took place in the after-school classroom at the participants’ school. The discussions in each group were moderated by researchers with a thorough background in the food sector. The discussions were recorded using mobile phones and notes were taken by an assistant in each group. The photographs of cheese dishes were shown to the team members 1 by 1. The same semi-structured interview guide was used in both groups.

The interview guide was organized as follows:

• What do you think of the appearance of the Chinese cheese dish? (for students)
• What do you think of the aroma of the Chinese cheese dish? (for students)
• What do you think of the taste or taste of the Chinese cheese dish? (for students)
• Do you like or dislike the Chinese cheese dish and why do you like or dislike it? (for students)
• What do you think about the challenges in cooking and the solutions? (for the canteen cooks)

### 2.6 Data analysis

The Kolmogorov-Smirnov test was employed to assess the normality of the data. The hedonic scores of sensory attributes conformed to normal distribution and the means and Std dev were calculated. A *t*-test was used to compare the means of two groups, Analysis of Variance (ANOVA) was applied to assess differences among three or more groups, and the Least Significant Difference (LSD) *post hoc* test was conducted to identify which specific groups differed from each other following a significant ANOVA result. Chi-squared tests of independence were used to detect differences in children’s recommended intentions to their parents between different types of cheese dishes. Lastly, principal component analysis was performed to identify the primary dimensions of consumer preference for cheese dishes. The recordings from the focus group discussions were transcribed verbatim and the analysis of the qualitative data was based on content analysis with pre-defined main themes. The results of the focus group discussions were systematically categorized into themes through Colaizzi 7-step analysis.

All quantitative analyses were conducted with SPSS (Version 24; IBM Corp., Armonk, NY, USA). The significance level was set at *P* < 0.05.

## 3 Results

### 3.1 Types of cheese dishes

During the study, 16 cheese dishes were made including 3 steamed, 2 ready-to-cook (only heating required), and 11 stir-fried dishes. The stir-fried dishes were classified as 3 types: paired with pure meat (1 dish), meat and vegetable (7 dishes), and vegetable (3 dishes). Table 1 presents examples of cheese dishes in each category of Chinese dishes. The nutrient content of 16 types of cheese dishes per 100g is provided in Supplementary Table 2.

**TABLE 1:** One example of the cheese dishes in each category of Chinese dishes.

Picture description	Example picture	Ingredients
Steamed dishes	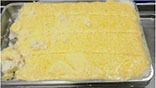	Rice Cheese
Ready-to-cook cheese dishes	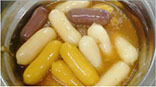	Rice cake Cheese
Pairing with pure meat (stir-fried)	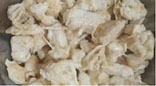	Chicken Cheese
Pairing with meat and vegetable (stir-fried)	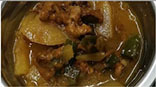	Pork Potato Pepper Cheese
Pairing with vegetable (stir-fried)	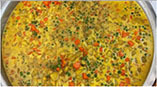	Pea Potato Carrot Cheese

### 3.2 Hedonic test

The mean and Std dev of appearance, aroma and taste of cheese dishes are listed in Table 2. For all the sensory attributes, ready-to-cook cheese dishes had a significantly higher score compared to those for steamed dishes and sir-fried cuisines (*P* < 0.05). The mean and Std dev of appearance, aroma and taste of the stir-fried cheese dishes are listed in Table 3. Among the sir-fried dishes, cheese paired with pure meat had a higher score compared to those of cheese paired with meat and vegetable, and with vegetable (*P* < 0.05).

**TABLE 2:** Hedonic test of the cheese dishes (M ± Std dev).

Sensory attributes	RCD	SD	SFD	*F*	*P*
Appearance	4.12 ± 1.15^a^	3.64 ± 1.43^b^	3.47 ± 1.33^b^	12.753	<0.001
Aroma	3.87 ± 1.27^a^	3.61 ± 1.50^a^	3.29 ± 1.38^b^	8.039	<0.001
Taste	3.82 ± 1.35^a^	3.53 ± 1.54^b^	3.28 ± 1.39^b^	7.712	0.001

M, mean; std dev, standard deviation. RCD, ready-to-cook cheese dishes; SD, steamed dishes; SFD, stir-fried dishes. Means with different letters within the same sensory attribute row are significantly different from each other (*P* < 0.05), as determined by post-hoc tests following a significant ANOVA result.

**TABLE 3:** Hedonic test of the stir-fried cheese dishes (M ± Std dev).

Sensory attributes	PPM	PV	PMV	*F*	*P*
Appearance	3.75 ± 1.33^a^	3.42 ± 1.34^b^	3.41 ± 1.31^b^	3.185	0.043
Aroma	3.61 ± 1.40^a^	3.20 ± 1.37^b^	3.28 ± 1.38^b^	3.656	0.027
Taste	3.60 ± 1.42^a^	3.20 ± 1.39^b^	3.26 ± 1.39^b^	3.406	0.034

M, mean; std dev, standard deviation. PPM, pairing with pure meat; PV, pairing with vegetable; PMV, pairing with meat and vegetable. Means with different letters within the same sensory attribute row are significantly different from each other (*P* < 0.05), as determined by post-hoc tests following a significant ANOVA result.

**FIGURE 2:**
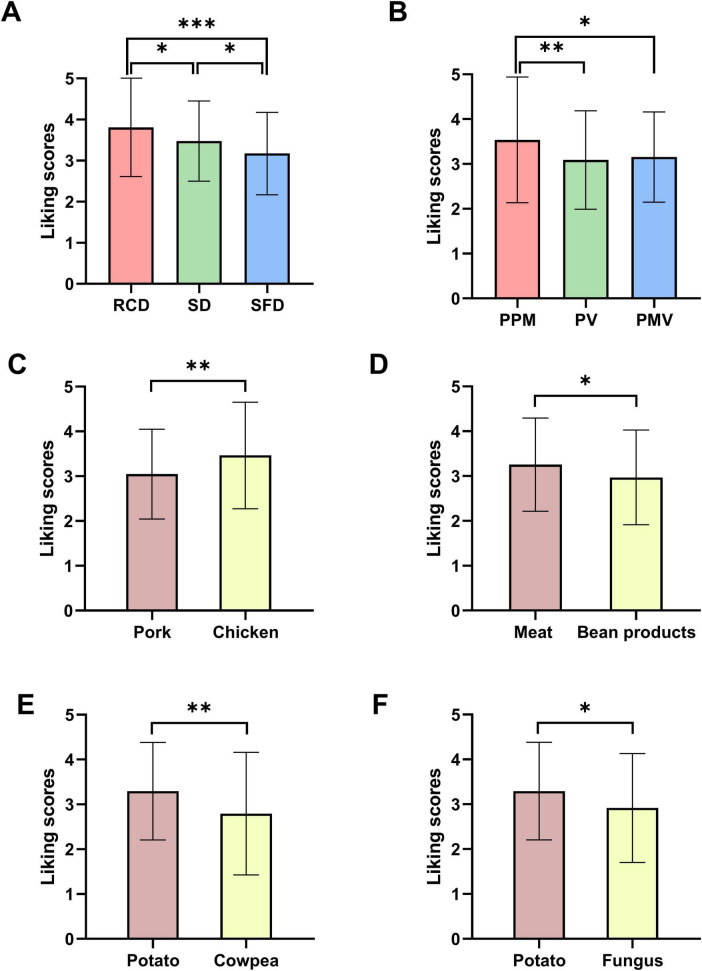
Difference comparison of cheese dishes’ overall liking scores. **(A)** Comparison of intergroup differences among RCD, SD, and SFD. RCD, ready-to-cook cheese dishes; SD, steamed dishes; SFD, stir-fried dishes; **(B)** Comparison of intergroup differences among PPM, PV, and PMV. PPM, pairing with pure meat; PV, pairing with vegetable; PMV, pairing with meat and vegetable. **(C)** Comparison of intergroup differences between pork and chicken. **(D)** Comparison of intergroup differences between meat and bean products. **(E)** Comparison of intergroup differences between potato and cowpea. **(F)** Comparison of intergroup differences between potato and fungus. Data are presented as the means ± standard deviations (*P < 0.05, **P < 0.01, ***P < 0.001).

The ANOVA results suggested that there was a significant difference in the overall liking scores of the 3 types of cheese dishes (*F*(2,118) = 10.777, *P* < 0.001). The LSD *post hoc* test determined the overall liking score of ready-to-eat dishes was higher than that of steamed dishes (*P* = 0.016), whereas that of steamed dishes was higher than stir-fried dishes (*P* = 0.028) (Figure 2A). The correlations between overall liking and sensory attributes were calculated, and the results showed that the ratings for overall liking were significantly correlated with the liking rating for each sensory dimension evaluated (appearance: 0.785, aroma: 0.898, taste: 0.892) (*P* < 0.001).

Among the stir-fried dishes, the overall liking scores differed for cheese dishes made from different raw materials. We found significant differences in the overall liking scores of cheese paired with meat, with meat and vegetable, and with vegetable (*F*(2,118) = 5.097, *P* = 0.007) (Figure 2B). Using an LSD *post hoc* test, cheese paired with meat was more popular than paired with vegetable (*P* = 0.003), and paired with meat and vegetable (*P* = 0.012). In the group paired with meat, the overall liking scores of cheeses paired with chicken was significantly higher than that of pork (*t* = 2.943, *P* = 0.004) (Figure 2C). In the group paired with high-quality protein food, the overall liking score of cheese paired with meat was significantly higher than that of soybean products (*t* = 2.115, *P* = 0.035) (Figure 2D). In addition, we found the overall liking scores of cheeses paired with potatoes was higher than that of cheese paired with cowpeas (*t* = 3.158, *P* = 0.002) (Figure 2E) and fungus (*t* = 2.545, *P* = 0.012) (Figure 2F) in the group of cheese paired with vegetable.

### 3.3 Recommend intention

**FIGURE 3:**
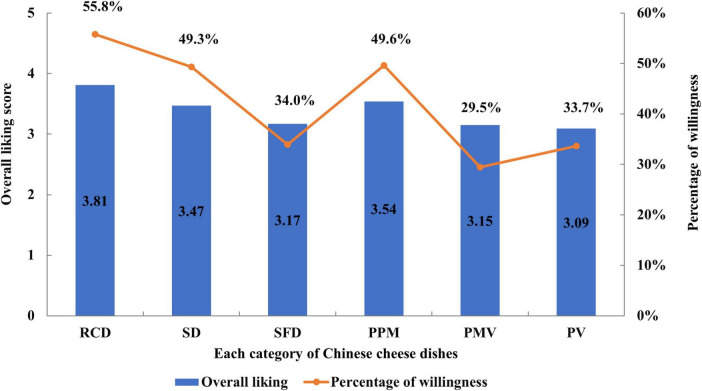
Percentage of willingness to recommend cheese dishes. Left ordinate represents score of overall liking and right ordinate represents percentage of recommend willingness. RCD, ready-to-cook cheese dishes; SD, steamed dishes; SFD, stir-fried dishes; PPM, pairing with pure meat; PMV, pairing with meat and vegetable; PV, pairing with vegetable.

The percentage of consumer intent to recommend to their parents based on sensory attributes are shown in Figure 3. A total of 55.79% of the children intended to recommend the ready-to-cook cheese dishes to their parents to buy or cook it at home, whereas 49.31% of the children were expected to recommend steamed dishes, and 33.96% to recommend the stir-fried dishes. The chi-square test of independence revealed significant differences in children’ recommend intentions across various types of cheese dishes. The ready-to-cook cheese cuisines and steamed cuisines were more likely to be recommend than the sir-fried dishes (χ^2^ = 12.777, *P* = 0.002). In addition, cheese paired with meat in the sir-fried dishes was more likely to be recommend than that paired with meat and vegetable, and with vegetable (χ^2^ = 12.279, *P* = 0.004). Moreover, the children were more likely to recommend dishes that had a higher overall liking.

### 3.4 Internal preference

**FIGURE 4:**
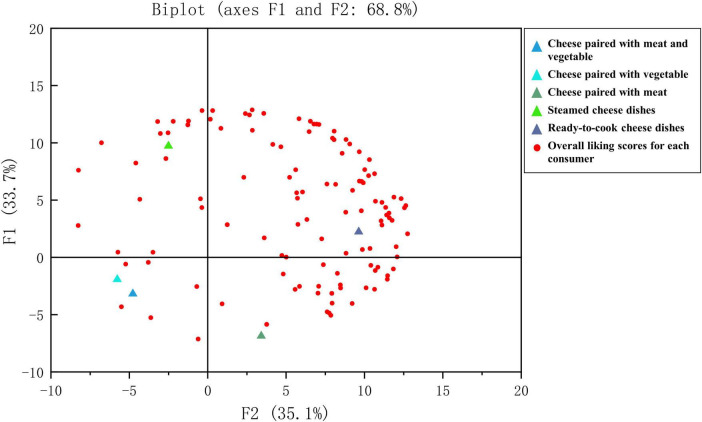
Internal preference mapping of different types of cheese dishes. The overall liking and consumer cluster means were regressed onto the consumer preference matrix generated by the principal component analysis (PCA). Dark blue triangle—cheese paired with meat and vegetable, light blue triangle—cheese paired with vegetable, dark green triangle—cheese paired with meat, light green triangle—steamed cheese dishes, and gray triangle-ready-to-cook cheese dishes. Red circles: Overall liking scores for each consumer.

The different types of cheese dishes and mean liking scores for each cluster were regressed onto the first 2 principal components of the consumer overall liking data to form an internal preference mapping (Figure 4). Principal components 1 (PC1) and 2 (PC2) explained 68.8% of the variation in the data. On the top-left side was the type of steamed dishes, On the bottom -left side were the types of cheese pairing with meat and vegetable, and pairing with vegetable. The type of ready-to-cook cheese dishes was on the top-right side, the type of cheese pairing with meat was on the bottom-right side. Moreover, the consumers are mainly scattered on the right side of PC2, which revealed the children consumers tended to prefer ready-to-cook cheese dishes.

### 3.5 Focus group discussion

Since cheese is a relatively new ingredient in Chinese cuisine, there was a process for canteen cooks to understand the characteristics of cheese and explore the cooking conditions. Simultaneously, there was a process for the consumers to accept and adapt these cheese dishes.

#### 3.5.1 Children’s evaluations of cheese dishes

The results of the children’s evaluations were consistent with the results of the sensory evaluation: ready-to-cook cheese dishes and steamed dishes were popular and likable products eaten as school lunches. The dishes that are already on the market, such as rice cakes with cheese and ready-to-cook foods, were rated more highly by the participants than the newly developed dishes in this study, especially stir-fried dishes. One of the participants mentioned that his family occasionally enjoyed having steamed rice with cheese in the evening at home. Steamed rice with cheese is delicious and full of flavor, whether consumed at home or in a school canteen. Another participant said, “It is delicious to put the rice cake with cheese into a hot pot for it is elastic and slightly chewy.” However, the attitudes of children toward stir-fried dishes were ambiguous and strongly correlated with the ingredients used; they preferred the dishes made from cheese with chicken, potatoes, soy products, vermicelli and meat rather than garlic sprouts, cowpeas, celery, fungus, carrots, gourd, and cabbage. One participant mentioned, “My top choice was the fried chicken with cheese. The chicken was scrumptious, and the inclusion of cheese enhanced its flavor without making it overly greasy.” Another participant commented, “Cheese and potatoes complement each other so well, delivering a sweet and creamy taste. I find myself craving an extra bowl of rice when I have this dish!”

#### 3.5.2 Canteen cooks’ experience

In this study, the canteen cooks were tasked with creating Chinese cheese dishes that incorporated the characteristics of cheese and the available ingredients in the canteens. Cheese is commonly used in baked dishes in Western cuisine, such as burgers, sandwiches, and pizza. However, since general school canteens do not have large ovens for baking, the closest cooking tool available is a steamer. Therefore, the canteen cooks experimented with adding cheese to steamed rice, steamed eggs, and steamed pumpkin, which are commonly served in the canteens. The chefs reached a consensus on the ease of cooking and the appearance and aroma of the dishes. However, the stretchiness of cheese posed a challenge in portioning out the meal.

In addition to these steamed dishes, the canteen cooks also experimented with adding cheese to some stir-fried dishes. However, since a large number of ingredients being stir-fried in a large pot leads to uneven heating, difficulty evaporating moisture inside food, and stick pot, this is major problem in school canteen cooking. Cheese is also rich in proteins that tend to aggregate and impact the texture. After encountering these issues, the canteen cooks had acquired valuable expertise through ongoing experimentation. To address these challenges, cheese was the last ingredient added to the pot and stirred quickly till slightly melted, the temperature was controlled to avoid high heat curdling the cheese in large chunks and affecting the taste. Regarding ingredient paired, the canteen cooks observed the effect of pairing cheese with numerous different ingredients and concluded that cheese is easier to stir fry with meat and has a good aroma and has a great flavor and bright color when paired with potatoes.

## 4 Discussion

In this study, 16 cheese dishes were innovatively created using traditional Chinese cooking methods in a school canteen. Through continuous refinement and adjustments to the recipes, students have a high overall acceptance of Chinese cheese dishes. Ready-to-cook cheese dishes were the most popular dishes among students, followed by steamed dishes, sir-fried dishes. Additionally, cheese paired with pure meat had the highest consumer acceptance from the perspective of food pairing.

Food choice is not 1-dimensional, but a complex human behavior influenced by many interrelating factors (20). Preferences are believed to be the primary factor influencing food choices, particularly when availability and economic factors are not involved (21). Many nutrition and epidemiological research studies have tried to understand consumer perceptions and food preference is influenced by factors such as familiarity, natural content, mood, convenience, sensory appeal (22). Among these possible factors, familiarity is related to the amount of previous exposure with the focal food, which is an important for determining people’s food preferences and choices because it decreases the uncertainty associated with the food and generates a better match between expectations and sensory characteristics (23, 24). In this study, cheese dishes cooked by the steaming method were more popular among students possibly because they appear more frequently in the market, similar to ready-to-cook foods. In other words, the children were already exposed to and more familiar with ready-to-eat foods such as rice cakes with cheese and steamed dishes such as baked rice with cheese before they participated in the study. Furthermore, repeated exposure to a particular food can increase the liking and consumption of a food that was formerly unappealing or even distasteful (25, 26). By quantitatively adding cheese cuisines to school lunches 3 times a week, we may be able to promote the habit of eating cheese for children and teenagers with the periodic exposure.

It is the actual eating experience that determines long-term consumption behavior, especially regarding sensory attributes (27). Our hedonic response to foods is a multisensory experience that depends not only on taste, but also on properties such as smell, appearance, and texture (28). In this study, the children’s liking of a Chinese cheese dish was assessed according to 3 different sensory modalities (taste, aroma and appearance) and there was a significant positive correlation between overall liking and liking of each sensory dimension. This is consistent with another study’s finding that “Individuals who have a greater preference for the sensory attributes of a food also tend to have a higher overall liking for that one” (29). Some studies have shown that sensory-based (visual, tactile, olfactory, taste) food education can increase children’s willingness to taste new foods, thus the preference for sensory attributes can also be cultivated in childhood (30, 31). Researchers also consider that the closer the odor-taste mixture is to the mental representation of the familiar food, the higher the level of pleasure for the taster (32). Different types of cheeses have different production processes, resulting in various cheese flavors. The hydrolysis of milk caseins, free amino acids and fatty acids, and the generation of sulfur-containing compounds are essential to the development of the cheese flavors (33). As a novel dish, it may be challenging for participants to feel familiar with the taste of cheese in Chinese cuisine. However, opting for cheese brands with more familiar flavors could potentially enhance the acceptance of Chinese cheese dishes. Mozzarella cheese constitutes nearly half of the cheese consumption in China’s food and beverage sectors, making it an excellent choice for introduction to the school lunch.

The preference test reflected different attitudes of the students when faced with the combination of cheese with different ingredients. When we added cheese to a meat dish, the school canteen had an easier preparation process and better student evaluations, such as fried diced chicken with cheese. Although the addition of cheese greatly increased the protein and calcium content of each dish, the diced chicken with cheese is the dish with the highest protein content among the 16 types of cheese dishes of the same portion. Protein is crucial in mimicking the taste of meat, which could imply a more appealing flavor for meat enthusiasts (34). However, for cheese paired with vegetable cuisines, the acceptance was reduced. Most students disliked vegetables other than potatoes, such as cowpeas, fungus, and rejected the pungent smell of garlic scape, particularly. Some cross-country studies have shown that potatoes and carrots are more popular in the vegetable category because potatoes are starchy and rich in carbohydrates, whereas carrots have a sweet taste and bright color. In contrast, green leafy vegetables such as cauliflower are strongly opposed because they are a little bitter and sometimes have a pungent odor (35, 36). To better promote Chinese dishes with cheese, we can start with ingredients that students prefer and combine cheese with meat or potatoes in a dish, then pair it with other vegetable cuisines to serve as nutritious lunches.

This study exhibited several notable strengths. Firstly, to our knowledge, this was the first attempt to make cheese dishes in a school canteen using Chinese traditional methods of cooking. Given that cheese is not typically utilized in traditional Chinese dishes, this study represents a pioneering effort. Second, the study can foster the practice of cheese consumption among children and adolescents to promote the physical and intellectual growth of students, which is a fabulous act. However, a few limitations need to be acknowledged. This study was a small pilot investigation limited to fourth-grade students, and the age as a potential factor influencing food preferences, was not considered in this analysis. To bolster the findings, future research should employ a more extensive and diverse sample. Furthermore, the sensory attributes assessed in the research survey were limited to overall evaluation of 3 aspects: appearance, aroma, and taste. A more detailed description and analysis of sensory attributes was not performed, such as further subdivision of taste properties into categories including mild, sweet, pungent.

## 5 Conclusion

Cheese was a harmonious complement to Chinese cuisine and was more readily embraced by students when steamed or integrated with meats. Introducing cheese into school lunches aids in fostering early cheese-eating habits among children and adolescents, enhancing the consumption of dairy products to align with dietary guidelines, and supporting their growth and development. Moreover, this initiative will pave the way for a flourishing cheese market and further promote the advancement of cheese within the Chinese market.

## Data Availability

The raw data supporting the conclusions of this article will be made available by the authors, without undue reservation.

## References

[1] Wang D Shinde S Young T Fawzi W. Impacts of school feeding on educational and health outcomes of school-age children and adolescents in low- and middle-income countries: A systematic review and meta-analysis. *J Glob Health.* (2021) 11:4051. 10.7189/JOGH.11.04051 34552720 PMC8442580

[2] Xia H Dai X Wang L Tian J Liang X Zhu W. Evaluation of micronutrient intake from nutrition lunch among primary school students. *Wei Sheng Yan Jiu.* (2016) 45:593–8. 29903328

[3] Huang Z Gao R Bawuerjiang N Zhang Y Huang X Cai M. Food and nutrients intake in the school lunch program among school children in shanghai, china. *Nutrients.* (2017) 9:582. 10.3390/nu9060582 28590431 PMC5490561

[4] Lin X Li Y Wu Q Lv Y Zhu Y Liu J Quality and quantity of school lunch in nanjing: based on data from the sunshine restaurant supervision platform. *Nubtrients.* (2024) 16:2184. 10.3390/nu16142184 39064627 PMC11280376

[5] Shi J Fang H Yu D Zhao L Ju L Guo Q. Consumption status of dairy products in Chinese children and adolescents aged 6∼17. *China Food Nutrition.* (2022) 28:26–30.

[6] Harper A Dobson R Morris V Moggré G. Fermentation of plant-based dairy alternatives by lactic acid bacteria. *Microb Biotechnol.* (2022) 15:1404–21. 10.1111/1751-7915.14008 35393728 PMC9049613

[7] Lorenzini E Lazzari B Tartaglia G Farronato G Lanteri V Botti S Oral ecological environment modifications by hard-cheese: from ph to microbiome: A prospective cohort study based on 16s rrna metabarcoding approach. *J Transl Med.* (2022) 20:312. 10.1186/s12967-022-03506-4 35810305 PMC9271248

[8] Alegría-Lertxundi I Rocandio P Arroyo-Izaga M. Cheese consumption and prevalence of overweight and obesity in a basque adult population: A cross-sectional study. *Int J Food Sci Nutr.* (2014) 65:21–7. 10.3109/09637486.2013.836741 24138541

[9] Alvarez-Bueno C Cavero-Redondo I Martinez-Vizcaino V Sotos-Prieto M Ruiz J Gil A. Effects of milk and dairy product consumption on type 2 diabetes: Overview of systematic reviews and meta-analyses. *Adv Nutr.* (2019) 10:S154–63. 10.1093/advances/nmy107 31089734 PMC6518137

[10] Guo J Astrup A Lovegrove J Gijsbers L Givens D Soedamah-Muthu S. Milk and dairy consumption and risk of cardiovascular diseases and all-cause mortality: Dose-response meta-analysis of prospective cohort studies. *Eur J Epidemiol.* (2017) 32:269–87. 10.1007/s10654-017-0243-1 28374228 PMC5437143

[11] Yang Y He M Cui H Bian L Liu J Cheng W Study on the incidence of lactose intolerance of children in China. *J Hygiene Res.* (1999) 28(1):46–8. 12712748

[12] Solomons N. Fermentation, fermented foods and lactose intolerance. *Eur J Clin Nutr.* (2002) 56(Suppl 4):S50–5. 10.1038/sj.ejcn.1601663 12556948

[13] Xu P Yang T Xu J Li L Cao W Gan Q Dairy consumption and associations with nutritional status of chinese children and adolescents. *Biomed Environ Sci.* (2019) 32:393–405. 10.3967/bes2019.054 31262385

[14] Rong Y Guo F Qu K Wang Y Lin R. Understanding Chinese consumer preferences for cheese products. *Food Sci Nutrition Res.* (2019) 2:2641–4295. 10.33425/2641-4295.1017

[15] USDA Foreign Agricultural Service, Global Agricultural Information Network. *Japan: Dairy and Products Annual.* (2021). Available online at: https://fas.usda.gov/data/japan-dairy-and-products-annual-7 (accessed October 10, 2024).

[16] USDA Foreign Agricultural Service, Global Agricultural Information Network. *South Korea: Dairy and Products Annual.* (2021). Available online at: https://fas.usda.gov/data/south-korea-dairy-and-products-annual-1 (accessed October 10, 2024).

[17] Ah J Tagalpallewar G. Functional properties of mozzarella cheese for its end use application. *J Food Sci Technol.* (2017) 54:3766–78. 10.1007/s13197-017-2886-z 29085119 PMC5643830

[18] Caldwell A Krause E. Mealtime behaviours of young children with sensory food aversions: An observational study. *Aust Occup Ther J.* (2021) 68:336–44. 10.1111/1440-1630.12732 33955028 PMC8363574

[19] Mueller C Zeinstra G Forde C Jager G. Sweet and sour sips: No effect of repeated exposure to sweet or sour-tasting sugary drinks on children’s sweetness preference and liking. *Appetite.* (2024) 196:107277. 10.1016/j.appet.2024.107277 38368909

[20] Chhabra I Kaur A. Development of a convenient, nutritious ready to cook packaged product using millets with a batch scale process development for a small-scale enterprise. *J Food Sci Technol.* (2022) 59:488–97. 10.1007/s13197-021-05031-6 35153306 PMC8814142

[21] Eertmans A Baeyens F Van den Bergh O. Food likes and their relative importance in human eating behavior: review and preliminary suggestions for health promotion. *Health Educ Res.* (2001) 16:443–56. 10.1093/her/16.4.443 11525391

[22] Głąbska D Skolmowska D Guzek D. Food preferences and food choice determinants in a polish adolescents’ covid-19 experience (place-19) study. *Nutrients.* (2021) 13:2491. 10.3390/nu13082491 34444648 PMC8400750

[23] Sick J Højer R Olsen A. Children’s self-reported reasons for accepting and rejecting foods. *Nutrients.* (2019) 11:2455. 10.3390/nu11102455 31615110 PMC6836127

[24] Torrico D Fuentes S Gonzalez V Ashman H Dunshea F. Cross-cultural effects of food product familiarity on sensory acceptability and non-invasive physiological responses of consumers. *Food Res Int.* (2019) 115:439–50. 10.1016/j.foodres.2018.10.054 30599962

[25] Laureati M Bergamaschi V Pagliarini E. School-based intervention with children. Peer-modeling, reward and repeated exposure reduce food neophobia and increase liking of fruits and vegetables. *Appetite.* (2014) 83:26–32. 10.1016/j.appet.2014.07.031 25106091

[26] Martins Y Pliner P. “ugh! That’s disgusting!”: Identification of the characteristics of foods underlying rejections based on disgust. *Appetite.* (2006) 46:75–85. 10.1016/j.appet.2005.09.001 16298456

[27] Ouyang H Li B McCarthy M Miao S Kilcawley K Fenelon M Understanding preferences for and consumer behavior toward cheese among a cohort of young, educated, internationally mobile chinese consumers. *J Dairy Sci.* (2021) 104:12415–26. 10.3168/jds.2021-20598 34482973

[28] de Araujo I Simon S. The gustatory cortex and multisensory integration. *Int J Obes (Lond).* (2009) 33(Suppl 2):S34–43. 10.1038/ijo.2009.70 19528978 PMC2726647

[29] Ju S Song S Lee J Hwang S Lee Y Kwon Y Development of nano soy milk through sensory attributes and consumer acceptability. *Foods.* (2021) 10:3014. 10.3390/foods10123014 34945565 PMC8701822

[30] Nekitsing C Hetherington M Blundell-Birtill P. Developing healthy food preferences in preschool children through taste exposure, sensory learning, and nutrition education. *Curr Obes Rep.* (2018) 7:60–7. 10.1007/s13679-018-0297-8 29446037 PMC5829121

[31] Reverdy C Chesnel F Schlich P Köster E Lange C. Effect of sensory education on willingness to taste novel food in children. *Appetite.* (2008) 51:156–65. 10.1016/j.appet.2008.01.010 18342395

[32] Fondberg R Lundström J Blöchl M Olsson M Seubert J. Multisensory flavor perception: The relationship between congruency, pleasantness, and odor referral to the mouth. *Appetite.* (2018) 125:244–52. 10.1016/j.appet.2018.02.012 29447997

[33] Forde A Fitzgerald G. Biotechnological approaches to the understanding and improvement of mature cheese flavour. *Curr Opin Biotechnol.* (2000) 11:484–9. 10.1016/s0958-1669(00)00130-0 11024368

[34] Flory J Xiao R Li Y Dogan H Talavera M Alavi S. Understanding protein functionality and its impact on quality of plant-based meat analogues. *Foods.* (2023) 12:3232. 10.3390/foods12173232 37685165 PMC10486508

[35] Dinnella C Morizet D Masi C Cliceri D Depezay L Appleton K Sensory determinants of stated liking for vegetable names and actual liking for canned vegetables: A cross-country study among european adolescents. *Appetite.* (2016) 107:339–47. 10.1016/j.appet.2016.08.110 27562674

[36] Estay K Pan S Zhong F Capitaine C Guinard JX. A cross-cultural analysis of children’s vegetable preferences. *Appetite.* (2019) 142:104346. 10.1016/j.appet.2019.104346 31278955

