# Supplementary material for: Exploring the acceptance of mozzarella cheese in school lunches among school-aged children: a pilot study
Source: Front Nutr. 2025 Jan 24;12:1495180. doi: 10.3389/fnut.2025.1495180 (PMC11804515; doi:10.3389/fnut.2025.1495180)
Supplement: Supplementary file 1 [file Table_1.docx]

| **Weekday** | **Staple Food** | **Dishes** | | | **Soup** |
| --- | --- | --- | --- | --- | --- |
| **Monday** | **Steamed Rice with Cheese** | **Braised Pork Balls** | **Scrambled Eggs with Tomatoes** | **Stir-fried Celery with Dried Bean Curd and Carrots** | **Spinach Soup** |
|  | Rice 100g, Cheese 35g | Pork 40g | Tomatoes 60g, Eggs 45g | Celery 50g, Dried Bean Curd 30g, Carrot 10g | Spinach 30g |
| **Tuesday** | **Black Rice** | **Chowder** | **Stir-fried Pork with Winter Squash** | **Stir-fried Cabbage with Broad Beans** | **Wax Gourd boiled Dried Shrimps Soup** |
|  | Rice 100g, Black Rice 30g | Quail Eggs 20g, Pork Rind 10g, Onion 15g, Fish Balls 20g, Carrots 10g | Winter Squash 40g, Carrots 20g, Lean Pork 10g | Broad Bean 20g, Tofu Skin 30g, Cabbage 40g | Wax Gourd 20g, Potherb Mustard 10g, Dried Shrimps 4g |
| **Wednesday** | **Lentil Rice** | **Fish Fillet** | **Scrambled Eggs with Sponge Gourd and Fungus** | **Stir-fried Pork with Green Pepper, Potato and Cheese** | **Radish Soup** |
|  | Rice 100g, Lentils 20g | Fish 50g | Sponge Gourd 40g, Carrot 15g, Eggs 20g, Black Fungus (Wet) 10g | Green Pepper 30g, Potato 30g, Lean Pork 10g, Cheese 35g | Radish 20g |
| **Thursday** | **Multigrain Rice** | **Braised Chicken Legs** | **Stir-fried Pork with Bottle Gourd and Fungus** | **Radish boiled Vermicelli** | **Tomato and Egg Soup** |
|  | Rice 100g, Grits (yellow) 30g | Chicken Leg 30g, Sesame Paste 10g | Bottle Gourd 50g, Black Fungus (Wet) 15g, Lean Pork 10g | Radish 50g, Vermicelli 10g, Red Pepper 15g, Dried Shrimps 6 g | Tomato 30g, Egg 15g |
| **Friday** | **Rice** | **Minced Pork with Tofu** | **Fried Chicken with Garlic, Potato and Cheese** | **Stir-fried Pumpkin** | **Red Bean and Sweet Soup Balls** |
|  | Rice 100g | Pork (Diced) 5g, Tofu 40g | Chicken Breast 10g, Garlic 40g, Potato 20g, Cheese 35g | Pumpkin 80g | Red Bean 10g, Glutinous Rice 20g |

Table S1 Lunch recipes for primary school students in the first week

Note: Dishes highlighted in red and bold indicate those that include added cheese. Fruit Supply for Week 1: 100g of oranges per student to be served after lunch on Tuesday. 100g of bananas per student to be served after lunch on Thursday.

Table S2 Analysis of nutrient content of 100g cheese dishes

| **Dishes’ name** | **Energy (kcal)** | **Protein (g)** | **Fat(g)** | **Carbohydrate(g)** | **Calcium(mg)** |
| --- | --- | --- | --- | --- | --- |
| Cheese Rice Cake | 205.54 | 5.10 | 2.80 | 39.40 | 131.20 |
| Cheese Bun | 191.92 | 10.20 | 14.30 | 5.90 | 399.50 |
| Steamed Rice with Cheese | 342.22 | 10.90 | 7.85 | 57.27 | 164.92 |
| Steamed Pumpkin with Cheese | 105.42 | 5.72 | 7.48 | 4.36 | 168.62 |
| Steamed Eggs with Cheese | 219.40 | 15.86 | 16.56 | 2.12 | 274.80 |
| Stir-fried Diced Chicken with Cheese | 211.57 | 23.22 | 12.88 | 0.93 | 181.96 |
| Kung Pao Chicken with Cheese | 206.57 | 13.51 | 14.03 | 7.11 | 228.72 |
| Fried Chicken with Garlic, Potato and Cheese | 197.06 | 11.61 | 14.50 | 5.81 | 247.72 |
| Stir-Fried Pork Slices with Cabbage and Cheese | 183.56 | 8.77 | 12.37 | 9.81 | 213.87 |
| Stir-fried Pork Slices with Loofah, Fungus and Cheese | 178.54 | 10.01 | 14.40 | 2.75 | 235.93 |
| Stir-fried Pork Slices with Cowpeas and Cheese | 172.71 | 9.92 | 13.35 | 4.72 | 234.89 |
| Stir-fried Pork with Green Pepper, Potato and Cheese | 168.09 | 8.18 | 11.19 | 9.42 | 162.80 |
| Stir-fried Pork Slices with Garlic Sprouts, Dried Tofu and Cheese | 196.26 | 12.71 | 14.42 | 4.64 | 269.33 |
| Stir-fried Vegetable Cubes with Cheese | 230.87 | 11.48 | 12.71 | 18.19 | 225.89 |
| Stir-fried Sponge Gourd and Beancurd with Cheese | 154.31 | 8.97 | 11.56 | 3.78 | 154.14 |
| Stir-fried Celery with Dried Tofu and Cheese | 193.09 | 11.22 | 15.26 | 3.09 | 319.86 |
